# Use of Laughter for the Detection of Parkinson’s Disease: Feasibility Study for Clinical Decision Support Systems, Based on Speech Recognition and Automatic Classification Techniques

**DOI:** 10.3390/ijerph191710884

**Published:** 2022-09-01

**Authors:** Miguel Terriza, Jorge Navarro, Irene Retuerta, Nuria Alfageme, Ruben San-Segundo, George Kontaxakis, Elena Garcia-Martin, Pedro C. Marijuan, Fivos Panetsos

**Affiliations:** 1 Neuro-Computing & Neuro-Robotics Research Group, Complutense University of Madrid, 28040 Madrid, Spain; 2 Innovation Group, Institute for Health Research San Carlos Clinical Hospital (IdISSC), 28040 Madrid, Spain; 3 Department of Economic Structure, CASETEM Research Group, Faculty of Economy, University of Zaragoza, 50009 Zaragoza, Spain; 4 Independent Researchers, Affiliated to Bioinformation and Systems Biology Group, Aragon Health Sciences Institute (IACS-IIS Aragon), 50009 Zaragoza, Spain; 5 Speech Technology Group, Information Processing and Telecommunications Center, 28040 Madrid, Spain; 6 Biomedical Image Technologies Group, Information Processing and Telecommunications Center, Universidad Politécnica de Madrid, 28040 Madrid, Spain; 7 Department of Ophthalmology, Miguel Servet University Hospital, 50009 Zaragoza, Spain; 8 Miguel Servet Ophthalmology Research Group (GIMSO), Aragon Health Research Institute (IIS Aragón), University of Zaragoza, 50009 Zaragoza, Spain

**Keywords:** machine learning, Parkinson´s disease, PD, biomarker, laugh, clinical decision support systems, automatic classification techniques, artificial intelligence

## Abstract

Parkinson’s disease (PD) is an incurable neurodegenerative disorder which affects over 10 million people worldwide. Early detection and correct evaluation of the disease is critical for appropriate medication and to slow the advance of the symptoms. In this scenario, it is critical to develop clinical decision support systems contributing to an early, efficient, and reliable diagnosis of this illness. In this paper we present a feasibility study for a clinical decision support system for the diagnosis of PD based on the acoustic characteristics of laughter. Our decision support system is based on laugh analysis with speech recognition methods and automatic classification techniques. We evaluated different cepstral coefficients to identify laugh characteristics of healthy and ill subjects combined with machine learning classification models. The decision support system reached 83% accuracy rate with an AUC value of 0.86 for PD–healthy laughs classification in a database of 20,000 samples randomly generated from a pool of 120 laughs from healthy and PD subjects. Laughter could be employed for the efficient and reliable detection of PD; such a detection system can be achieved using speech recognition and automatic classification techniques; a clinical decision support system can be built using the above techniques. Significance: PD clinical decision support systems for the early detection of the disease will help to improve the efficiency of available and upcoming therapeutic treatments which, in turn, would improve life conditions of the affected people and would decrease costs and efforts in public and private healthcare systems.

## 1. Introduction

**Figure 1:**
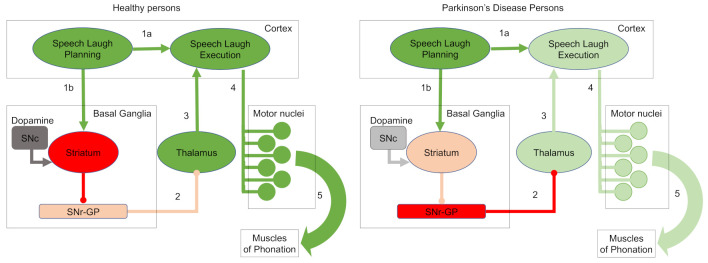
Simplified representation of how Parkinson’s disease affects speech and laughter. Speech/laughter decision-making cortical areas activate the motor commands–execution circuit (**arrow 1a**) as well as the basal ganglia–thalamus circuit (**arrow 1b**), which modulates the activity of these commands (**arrow 3**). Motor commands–execution areas send their output (**arrow 4**) to the motor nuclei which control muscles that generate speech/laughter sounds (**arrow 5**). In green, excitatory neuronal activity; in red, inhibitory neuronal activity; in grey, activity of dopaminergic neurons. Intense color indicates high neuronal activity; light color indicates low neuronal activity. In healthy subjects (**left scheme**), SNc-produced dopamine excites striatum neurons that inhibit SNr-GP inhibitory neurons. Low inhibitory input to the thalamus (**arrow 2**) is the ideal condition for the correct modulation of the motor commands (**arrow 3**), as well as the coordination of the motor nuclei (**arrow 4**) and of the corresponding muscles (**arrow 5**). In Parkinson’s disease (**right scheme**), the reduced SNc dopamine slows down striatum neurons, increasing SNr-GP inhibitory output (**arrow 2**). The inhibited thalamus fails in the modulation of cortical nuclei (**arrow 3**), losing the coordination of the motor nuclei (**arrow 4**) and provoking motor disorders (**arrow 5**). SNc, substantia nigra compacta; SNr, substantia nigra reticulata; GP, globus pallidus.

Parkinson’s disease (PD) is a neurodegenerative disorder, the main pathological characteristic of which is degeneration of the cells of the substantia nigra (SN) that produce dopamine. The drop in the level of dopamine causes the onset of typical motor symptoms (Figure 1) [1,2]. PD is characterized by a wide range of clinical features which include both motor and non-motor symptoms [3]. Regarding motor symptoms, PD patients express bradykinesia/akinesia, rigidity, postural instability, and rest tremor. Akinesia is the difficulty of initiating a movement; it causes a decrease of the voluntary acts, and it is often associated with bradykinesia, a slowdown of the speed of movements. PD is the most common neurodegenerative disease after Alzheimer’s, with over 10,000,000 cases worldwide, and high associated social and economic burdens that reached $52 billion in the USA and €14 billion in the EU. Male patients’ incidence rate is twice as high as females’ [4].

Clinical decision support systems for the evaluation of neural PD damages are based on biomarkers like motor, functional, and behavioral alterations of the patient [4,5]. However, PD motor symptoms are not only limited to upper and lower limb movements; they also affect mouth articulation and laryngeal muscles coordination [6]. Indeed, throughout the course of the disease, 90% of patients develop “hypokinetic dysarthria”, a disorder characterized by volume and pitch variation in their voice, inconstant speech rate, imprecise articulation of the consonants, presence of breath noise, as well as lack of coordination or even paralysis of speech mechanisms, which in turn affect phonation, articulation, and prosody [7]. Thanks to the powerful signal processing technology, very fine speech alterations have been identified in PD patients: articulation abnormalities [8,9], phonation variations, reduction of fundamental frequency variability, etc. [10,11].

However, speech alterations by themselves cannot be used as PD biomarkers since several studies reported their ineffectiveness for the detection of the disease [12,13]. Performances can improve by using more complex features to parametrize speech signals, also combined with machine learning techniques, similar to those used in speaker recognition problems [11,14,15]. However, none of these clinical decision support systems is oriented to the accurate detection of the disease.

Laughter carries a significant amount of information [16], has long been considered a depression biomarker, and has been postulated as a candidate for the detection of other neurological disorders [17]. Furthermore, laughter is differentially affected by the diverse neurological disorders [18,19], which could make it useful in the discrimination of common syndromes (e.g., PD dementia) [20].

Based on the primitivity of laughter, we hypothesize that laughter-based systems could be more effective than speech-based ones for accurate detection of PD. Since laughter is a more primitive and less elaborate sound expression than speech, we expect subtle changes, normally covered by the complexity of the speech signals, to be detected. Indeed, we know from anatomical and physiological data that, for sound expression, speech and laughter processes share the same laryngeal, respiratory, abdominal, and maxillofacial muscles and joints [21], and that laughter is a primitive sound expression, less complex and less subject to voluntary control than speech [21,22]. Therefore, PD-originated motor dysfunctions will cause laughter alterations similar to speech ones. On the other hand, laughter has been proved to be a valid biomarker for decision support systems in diagnosis and evaluation of diseases involving motor syndromes, like depression [23]. Some speech recognition techniques have been used in PD patient identification with over 80% success rate [24,25,26]. Based on these premises, we hypothesize that PD-originated laughter alterations can be detected by means of speech recognition techniques.

**Figure 2:**
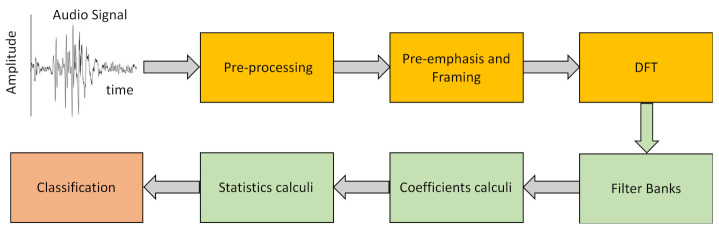
Temporal representation of one of the signals used in the study, followed by the steps of the analysis pipeline. DFT, digital Fourier transform. “Filter Banks” include Mel, Human Factor, and Bark filters.

In the present paper, we provide evidence for the feasibility of clinical decision support systems for the accurate diagnosis of Parkinson’s disease based on the acoustic characteristics of laughter, analyzed with speech recognition methods, and categorized with automatic classification techniques. Following the scheme of Figure 2, laughs are preprocessed, and a database of laugh signals is created. Each laugh is framed (divided into small, partially overlapping windows) and power spectra are obtained by means of a Fourier transform. Then, each laugh is associated with a set of coefficients, real numbers representing specific changes in the frequencies of this laugh obtained by passing the signal through a set of simple filters. Part of the laughs dataset (laughs now represented by their corresponding coefficients) is used to train an automatic classification system and divide laughs as PD or non-PD. The performance of the automatic classification system is tested using the rest of the laughs in the dataset, that is, laughs not employed in the phase of training.

## 2. Materials and Methods

### 2.1. Laughter Recordings and Preprocessing

Individual laughs (N = 120), 60 corresponding to healthy subjects and 60 corresponding to PD-suffering patients (equally divided between sexes), were extracted using Audacity [27] from recording sessions in which subjects were watching humoristic videos. Original audios were sampled at 44.1 KHz, then digitized at 16 bits and downsampled at 16 KHz. All subjects gave detailed consent to participate in this study, which was conducted in accordance with the guidelines established by the Ethics Committee of the Miguel Servet Hospital and based on the principles of the Declaration of Helsinki. The experimental protocol was approved by the local Ethics Committee (CEICA: Ethic Committee of Clinical Research of Aragon, Spain). Laughs were obtained from a clinical trial performed by the Aragon Institute of Health Science (IACS), Zaragoza, Spain. The Ethics Committee of Aragon revised and approved the clinical protocol of the study. The diagnosis of PD was based on standard clinical and neuroimaging criteria [28] and information about disease severity using the Hoehn Yahr scale [29]. Disease duration and treatment were recorded. Disease duration in the group of patients at the beginning of the study was 13.56 years (SD = 6.22). The median Hoehn Yahr stage at the beginning of the study was 2.68 (SD = 0.69). These are patients with early or moderate disease duration and severity.

### 2.2. Laughter Characterization Using Speech Recognition Techniques

Each laught was characterized by means of a vector of cepstral coefficients, i.e., mathematical identifiers containing information about signal changes in different spectrum bands [30]. The use of cepstral coefficients is very popular and commonly used in speech recognition problems [31]. The main advantage of audio characterization by cepstral coefficients is that we can separate the signal into two components, one corresponding to the source (vocal cavities, glottis, mandible, etc.) and the second to the speaker, without any a priori knowledge about the source [32]. Before cepstral coefficient analysis, signals are passed through non-linear scaled filters to mimic human pitch perception.

### 2.3. Cepstral Coefficients

Mel frequency cepstral coefficients (MFCCs) are one of the most frequent representations of a sound in speech recognition techniques. They are based on a linear cosine transform of a log power spectrum on a nonlinear Mel frequency scale, which resembles the psychoacoustic behavior of the human ear.

MFCCs are obtained by means of a bank of triangular band-pass filters which convert the linear power spectrum on a logarithmic scale, the Mel scale [33].

To build our decision support system we have evaluated the performance of the classical MFCCs as well as two very common variations, Mel human factor cepstral coefficients (HFCCs) and Bark frequency cepstral coefficients (BFCCs) [33,34]. The three types have been employed in speech recognition-based PD decision support systems [25,33]. HFCCs are extracted using a Mel scale filter bank, the bandwidth of which varies according to the expression of the equivalent rectangular bandwidth (ERB). BFCCs employ a combined frequency representation of the acoustic signal, linear below 500 Hz and logarithmic above. Furthermore, unlike MFCCs, BFCCs employ a greater bandwidth for the higher frequencies.

All coefficients were extracted from laugh signals, both from healthy people and from people with PD, using the generic extraction method and different banks of 26 filters. Normally the number of filters used varies between 20 and 40, with 24 and 26 being the most used [34].

### 2.4. Laughter Processing

The calculation of the different cepstral coefficients was carried out in seven steps, implemented in Matlab R2019a [35].

***Pre-Emphasis.*** *The objective of this step is to compensate for the filtering effects exerted by the glottis and the vocal tract on the signal by enhancing the value of the higher frequencies. For this, a high-pass FIR filter (1) is applied to the original signal*

(1) $$H\left(z\right)=1-{\mathrm{kz}}^{-1},\;0\;<\;k\;<\;1$$

*where H(z) is the amplitude difference between the output and the input of the filter, expressed in terms of Z-Transform. At higher k-values the attenuation of the low frequencies is greater. Here we have used k-values between 0.95 and 0.98 to attenuate DC offset, electrical noise, etc. Pre-emphasis filter corresponds to a first order high-pass filter. For C1 = k filter modifications, cut-off frequency (in this case 1840 Hz) is maintained; maximum attenuation is being modified for lower frequencies while increasing “k”.*

***Framing–Windowing.*** *To process an acoustic signal that is continuously changing with time, the original signal is divided into very short segments in which we can assume that its characteristics are static. Further, we employ window overlapping to avoid large variations between the segments to be analyzed, this overlap being less than the size of the selected windows. In a preliminary analysis we have shown that laugh signals can be considered invariant in intervals of duration less than 30 ms. For our study we have used 25 ms-long windows with 10 ms inter-window overlap.*
***Discrete Fourier Transform (DFT).*** *After framing, the power spectrum of each window is calculated using Equation (2).*

(2) $$X_{k}=\overset{N-1}{\underset{n\;=\;0}{\sum }}x_{n}{\;*\;e}^{-j\left(\frac{2\pi }{N}\right)\mathrm{kn}}$$

*To reduce edge effects during DFT (distortions at the edges of the signal generated by the convolution of finite duration/length signals) we previously applied a Hanning window which reduces side lobe level amplitude.*

***Filter banks.*** *We have used different filter banks, one for each type of cepstral coefficients. In the case of Mel scale filters, the scale of the power spectrum is transformed into a non-linear scale (Mel scale). For this, the power spectrum is multiplied with the Mel scale filter bank. This transformation is given by Equation (3).*

(3) $$f\left(\mathrm{mel}\right)=1125\;*\;\ln\left(1+\frac{f\left(\mathrm{Hz}\right)}{700}\right)$$

*In the case of human-factor filters, the power spectrum of the signal is transformed to the Mel scale as above but, in this case, the relationship between bandwidth and the central frequency of each filter is corrected through the expression of the equivalent rectangular bandwidth (ERB) given by Equation (4) as a function of the central frequency (f_c_).*

(4) $$\mathrm{ERB}\left(f_{c}\right)=6.23f_{c}{}^{2}+93.39f_{c}+28.52\left(\mathrm{Hz}\right)$$

*In the case of Bark scale filters, the power spectrum of the signal is transformed into the Bark spectrum by passing the DFT through a series of filters corresponding to the Bark scale. The change in scale is given by Equation (5).*

(5) $$f\left(\mathrm{Bark}\right)=13{\;*\;\tan}^{-1}\left(\frac{0.00076*f\left(\mathrm{Hz}\right)}{1000}\right)+3.5{\;*\;\tan}^{-1}\left({\left(\frac{f\left(\mathrm{Hz}\right)}{7500}\right)}^{2}\right)\;$$

***Discrete cosine transform (DCT).*** *Cepstral coefficients are calculated by computing the DCT of the log-spectrum of the signal obtained after passing through the corresponding filter bank given by Equation (6).*

(6) $$c\left(n\right)=\sum_{m\;=0}^{M-1}\log_{10}\left(s\left(m\right)\right)\;*\;\cos\left(\frac{\pi n\left(m-0.5\right)}{M}\right)$$

*with s(m) being the power spectrum of the signal after passing through the filter, “m” the m-st filter (m = 0 to M), and “n” the n-st coefficient (n = 0 to N). For speech recognition, 12 to 20 coefficients are used, with 13 being the most used since more coefficients provide redundant information and adds complexity to the systems [34]. In our study we employed 26 filters (M = 26).*

***Laugh characterization.*** *With the above procedure we obtain 13 cepstral coefficients for each of the T frames we divide each laugh into, with T being a high number that depends on the duration of the record. To characterize the laugh, we calculate the mean (μ_i_) and the standard deviation of the mean (SD_i_) of each of the 13 coefficients for the whole record (i = 1 to 13). However, cepstral coefficients only represent static characteristics of the signal since the T frames of the signal are assumed to be static. To include dynamic information, we additionally calculate Δc and ΔΔc, the first- and second-order variations of the extracted coefficients for each of the T frames using Equations (7) and (8) [36].*

(7) $$\Delta c\left(n\right)=\frac{c\left(n+1\right)-c\left(n-1\right)}{2}$$

(8) $$\Delta \Delta c\left(n\right)=\frac{\Delta c\left(n+1\right)-\Delta c\left(n-1\right)}{2}$$

**Figure 3:**
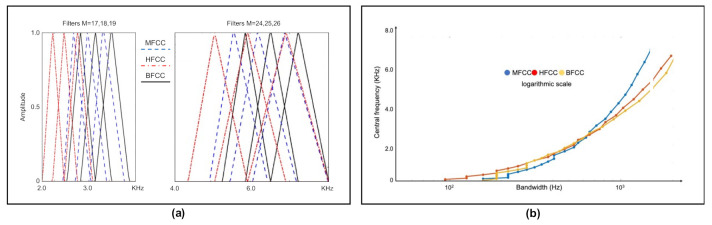
(**a**) Representation of 6 filters corresponding to each bank, with lower M corresponding to filters with lower central frequency. At lower frequencies, the Bark and HFCC filters have a lower bandwidth; this bandwidth increases in relation to the filter’s central frequency, which is higher for higher frequencies. The bandwidth of the MFCC corresponds to [$fc_{m-1},fc_{m+1}]$. The center frequencies of the filters correspond to those of Table 1. (**b**) Relation between bandwidth and central frequency of the filters in a logarithmic scale. Points correspond to filter M = 1:26.

*Following what we did with the cepstral coefficients we calculated the mean (μ_i_) and the standard deviation of the mean (SD_i_) of each of the 13 coefficients Δc_i_ and ΔΔc_i_ for the whole record (i = 1 to 13). This way, each laugh signal is finally identified by a unique 78 component-long vector whose values are the means and the standard deviations of the 13 cepstral coefficients, the 13 Δc and the 13 ΔΔc of this signal or by the 176 if also kurtosis and skewness are added (see Figure 3).*

**Figure 4:**
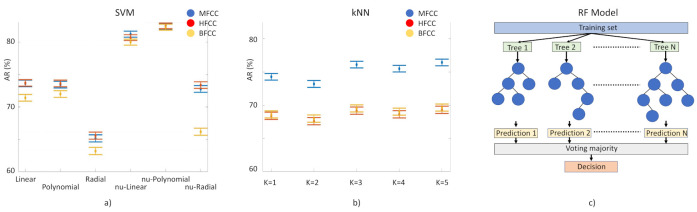
(**a**) SVM performance as a function of kernel selection. (**b**) kNN performance as a function of k, k = 1,5. AR with 90% confidence interval in both cases (Blue, MFCC. Yellow, BFCC. Red, HFCC). (**c**) Graphic representation of the RF model. Training data set is split into N different data subsets that feeds into the N generated decision trees (N = 100 in our study). Decision is taken following the final prediction, obtained by majority voting of the N decision trees, weighting the models according to their performance.

***Laughter classification using automatic classification techniques.*** *For the identification of PD laughs for our decision support system, we have tested the performance of three supervised learning-based classification techniques: (1) Random Forest (RF) model based on the generation of T decision random trees [37]. We execute it 100 times, without pruning. (2) Classification method, kNN [38]. Input elements are represented as vectors and for each one of them the Euclidean distance with each of its k closest neighbors is calculated. Here we tested k = 1 to 10. (3) Support Vector Machine (SVM) [39]. This separates the two classes to be predicted by means of a hyperplane. In this case, we have used a linear kernel, based on previous studies in which this method has been used with MFCC coefficients with successful results. We have used ν-SVC [40] as the SVM type, in such a way that there is a margin of error, upper bound and lower bound, between the examples that may fall into the opposite plane in training: this value has been set at 0.5. Several kernels have been tested: linear, polynomial 3rd degree, radial basis, ν-linear, ν-polynomial 3rd degree, ν-radial basis (Figure 4, left). The 156 component-long characteristic vectors of the laughs were employed as input vectors for these classification methods using the function implemented in WEKA [41]. Models need to be trained to tune up their parameters and then to be validated for the evaluation of their performance. For training and validation, we used subject-wise k-fold cross-validation. This method is based on splitting the dataset in k segments; at every iteration, k-1 segments are used for training and one for validation (Figure 4, right).*
***Overall performance of cepstral coefficients.*** *The performance of the three different types of cepstral coefficients in the machine learning models was evaluated according to the accuracy rate (AR), validated through the Mathews correlation coefficient (MCC). Overall performance of the laugh identification-and-classification procedure, expressed by the AR, which represents the percentage of correct predictions given by Equation (9):*

(9) $$AR\left(\%\right)=\frac{Number\;of\;correct\;predicted\;examples}{Total\;examples}*100$$

*The highest score was obtained by the RF when fed with MFCC with AR = 83%. In the second place we find the SVM fed with HFCC with AR = 83%. The kNN algorithm performs worse (76% AR, with MFCC) and for this reason it has been excluded from further consideration for implementation in our decision support system. However, kNN behaves in a very stable way, showing AR values over 66% (over 70% with MFCC) with k = 1 to 5.*

**Figure 5:**
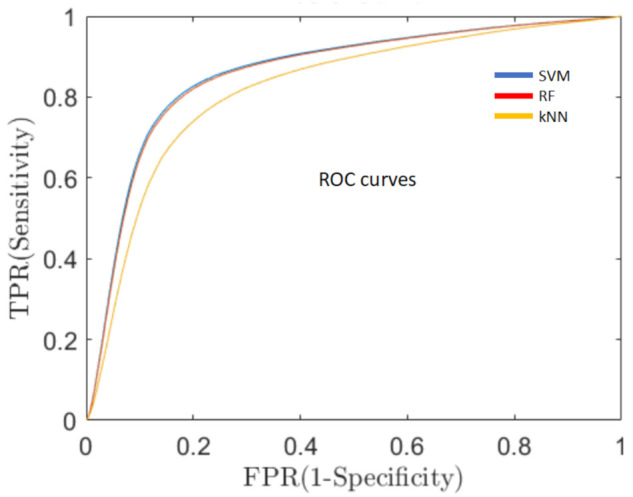
Representation of the receiver operating characteristics (ROC) curve for the three cepstral coefficients (MFCC, HFCC and BFCC) with the best performances. ROC relates the true positive rate (TPR) with the false positive rate (FPR) and the area under the curve (AUC) that measures the degree of separability between the two classes. Blue line represents an SVM with a ν-polynomial kerne, BFCC filter bank. Red line corresponds to an RF, MFCC filter bank. Yellow line corresponds to a kNN, k = 5, MFCC filter bank. 10-fold validation.

*We validated AR results through the Mathews correlation coefficient (MCC), a very good measure method employed in machine learning techniques [1]. MCC is the Pearson’s (or Yule’s) φ coefficient which measures the accuracy of a binary classification [42]. It is calculated from the confusion matrix, and it takes values between 0 and 1, with 0 a random prediction and 1 a perfect prediction [43]. MCC results are in agreement with AR results: the highest score was obtained by RF fed with MFCC, MCC = 0.66 and in the second place we find the SVM fed with HFCC, MCC = 0.64.*
***Sensitivity of the classification algorithms.****A good overall performance, a high AR, is a necessary but not sufficient condition for the development of a clinically useful decision support system. One of the fundamental requirements is to minimize the percentage of false negative predictions (ill persons classified as healthy), thus, reducing the number of PD patients that could not be detected and, consequently, would not receive early medical care. For this reason, in addition to AR, we evaluated the sensitivity of the system, which means the capacity of the system to classify true PD patients as having PD, by means of the receiver operating characteristics curve (ROC, Figure 5). ROC is a probability curvethat relates the true positive rate (TPR), i.e., PD subjects correctly classified as PD patients, with the false positive rate (FPR), i.e., healthy subjects erroneously classified as PD ones, at various threshold settings. One of the most important metrics of the ROC curve is the area under the curve (AUC) that measures the degree of separability between the two classes (healthy and PD) [44]*.

## 3. Results

A good clinical decision support system should not commit errors in the identification of true PD or, at least, they should minimize the number of such errors (high sensitivity—TPR). On the other hand, if we must choose between a low rate of false positive and a low rate of false negative identifications (healthy subjects classified as PD and PD subjects classified as healthy, respectively), for a clinically useful support system, the second choice is mandatory. Following these criteria, we chose to build the clinical decision support system by coupling the cepstral coefficients with an RF classification procedure.

Finally, we evaluated the performance of the clinical decision support system on a dataset of 20,000 laughs of both sexes, randomly generated from healthy and PD subject laughs. None of these laughs was employed in the cepstral coefficients selection nor in the training or testing of the decision support systems. Random laughs of each type were generated with the same M and STD of the corresponding real laughs. Random laughs are generated by means of the “mvnrnd” function from Matlab, which generates normal multivariable random numbers. This function, represented as R = MVNRND(μ,σ,N), returns the N × D R matrix, where N represents the population and D the extracted features of randomly chosen vectors from the multivariate normal distribution with mean vector $\underset{\mu }{\to }$ and covariance matrix generated by means of the variance of each feature. $\underset{\mu }{\to }$ is a 1 × D vector and σ is a D × D symmetric matrix. For the generation of the laughs, $\underset{\mu }{\to }$ and σ are obtained from the original post-processed laughs, which means from the statistical values of their coefficients. A numerous second data set of real laughs could be used.

Results are exposed in Table 2, Table 3 and Table 4.

Both, RF- and SVM-based clinical decision support systems reached 81–83% AR with the three filters (Table 5 and Table 6) with 0.85–0.86 AUC values, suggesting that cepstral coefficients are generally good for classification, regardless of the employed algorithm (RF or SVM). This is especially important because one can gain much interpretability using, for example, a linear SVM (by examining the weights of the classifier), without incurring a greater rate of false negatives.

To determine to which extent our classification is affected by laugh’s pitch characteristics (power spectra), we employed it instead and in addition to the cepstral coefficients as an input to our classification system. In both cases, pitch information is not a determinant for the correct classification of the laughs, as AR is very low when pitch statistics (mean, standard deviation, etc.) were employed as input attributes (AR < 50%).

## 4. Discussion

In the present paper we provided evidence for the feasibility of a clinical decision support system for the detection of Parkinson’s disease which employs laugh as a biomarker of the illness. Such a decision support system would be composed by two sub-systems: one for laugh identification and one for laugh classification.

For the first, we tested the suitability of 13 cepstral coefficients, together with their delta and delta-delta components, employing three different filter banks (Mel, Bark and Human), each of which is composed by 26 filters. For the second, we tested three automatic classification techniques (kNN, RF and SVM). Each of them was tested three times; one for each of the three coefficients.

We proved that classical speech-recognition techniques like cepstral coefficients can be used to identify and label laugh signals and that such coefficients can be used by automatic classification techniques to decide if laughs belong to a PD or non-PD subject. All of them reached very good AR scores, the highest (83) obtained through the clinical decision support system based on the RF classification model using the Mel cepstral coefficients. This model has been used for the final test due to the lower computational cost compared to the SVM. As mentioned in the Results section, SVM performed similarly. High AR scores have been obtained using both Bark and Human Frequency cepstral coefficients in the final test, proving the consistency of our approach. Mathews correlation coefficient (MCC), an independent measure of the accuracy of the classification, corroborates the best AR performance of RF and SVM models, allowing them 0.66 and 0.64 points over 1.0 scores, respectively. A limitation of the study is that testing has not been performed on a data set of real laughs.

The similar and high AR values obtained by the RF, when combined with Human Factor or Bark frequency cepstral coefficients, prove the consistency of the approach, and suggest the models are comparable. Metrics displayed in Table 2, Table 3, Table 4 and Table 5 indicate that, on one hand, individual moments do not carry enough information for a correct classification of the subjects and, on the other, we constantly improve classification performance if we consider these moments in an incremental manner.

In SVM data obtained with the three kernels, we observe that linear and polynomial kernels achieve similar ARs, higher than AR of the radial one, which suggests that clusters are not formed by partially intermingled clouds and that they can easily be separated by simple planes.

Pitch contribution in the correct classification of the laughs was also tested. Laugh presents a high fundamental frequency variation [35]. This variability is present in all groups and sexes, making fundamental frequency non-suitable as a feature for laugh-based PD classification (AR < 50% when pitch statistics were employed as the sole input attributes). However, pitch does not provide relevant information for classification performance, since classification systems do not improve their AR. This is possibly due to a very low contribution of vibrational components in the characterization of laugher signals, contrary to what occurs in speech ones. Power spectra represent the vibrational components of the signal, which, in our case, are generated by the vocal apparatus during sound production. In neural circuits terms, these results could indicate that laugh analysis primarily detects the degeneration of specific motor nuclei and the reduction of the precise control they exercise to the muscles through the laryngeal reflexogenic control systems [45,46] instead of the degeneration of higher brain areas, like basal ganglia, thalamus or cortex and the global control each of them exercises to the next one (Figure 1), which would also include significant deterioration of the vibrational components. Other not mutually exclusive interpretations are possible, as for example the PD-independent influence of sex on pitch.

Our results are consistent with automatic Parkinson’s disease detection systems using speech analysis with MFCC that have obtained AR values higher than 80% [25]. The interest of laugh-based clinical decision support systems we propose could be useful for early detection of the disease, where motor symptoms are not yet detectable by neurologists and early detection of neurodegenerative diseases could facilitate treatments to slow down the evolution of the illness.

From a computational point of view, we could highlight that, a priori, the decision support system does not display significant AR differences depending on the selection of the filter bank. This provides relevant information for future studies in laughter-based PD detection since the development of MFCC algorithms is very extended and numerous libraries with their implementations can be easily found. Open-source libraries are available, like Librosa for Python or OpenSmile, where the Mel filter bank is applied by default. On the other hand, Matlab’s Audio Toolbox provides an MFCC extraction function, with an approximate cost of less than 700€ for an annual license.

However, the study of the coefficients themselves should be expanded, by evaluating the number of employed filters as well as the number of coefficients, to achieve a compromise between optimal results and computational cost penalty. Furthermore, the adjustment and evaluation of SVM hyper-parameters would be of interest for future studies to further understand input features. Possibly, neural networks and deep learning techniques, would help to build the decision support system for clinical use.

In future studies the variability of the humoristic videos and the psychological conditions of the subjects should also be considered, as well as the possible high variability in laughter production, and even that some of the subjects could not feel comfortable during the recording. The possible combination of speech and laugh analysis to improve PD detection performance could facilitate the implementation of a system for the telematic detection of PD. Also, the possibility of evaluating the process of the disease would be of interest, trying to estimate the UPDRS (Unified Parkinson’s Disease Rating Scale) scale of PD patients through speech and laughter, with a more continuous evaluation of the disease and a consequent reduction of health costs. Smartphone apps could be useful for allowing people to perform the test in privacy, thus improving the above-mentioned aspects.

## 5. Conclusions

Our paper provides evidence that (1) laughter can be used as a biomarker for PD detection, (2) laughter-based support systems are feasible, and (3) laughter-based support systems perform at least as well as speech-based ones, thus giving PD specialists the possibility to perform a prospective study of laughter recordings from people who eventually develop PD. As demonstrated in our experiments, the feature extraction methods (cepstral coefficients) and machine learning algorithms derived from speech processing field can provide promising results for PD detection from laughs.

The main contributions of our study are to have proven the feasibility of using laughter as a possible biomarker to detect Parkinson’s disease and having used speech analysis techniques on much more primitive signals such as laughter.

## Figures and Tables

**Table 1:** Central frequencies corresponding to each of the 26 filters for the three scales employed in this study: Mel, Human Factor, and Bark.

Filter Nr	Mel (MFCC)	Human Factor (HFCC)	Bark (BFCC)
1	62.50	31.25	62.50
2	156.25	125.00	156.25
3	218.75	187.50	218.75
4	312.50	281.25	312.50
5	406.25	375.00	375.00
6	531.25	468.75	468.75
7	656.25	593.75	562.50
8	781.25	718.75	656.25
9	937.50	843.75	750.00
10	1093.75	1000.00	875.00
11	1250.00	1156.25	1000.00
12	1437.50	1343.75	1156.25
13	1656.25	1531.25	1281.25
14	1875.00	1781.25	1468.75
15	2125.00	2000.00	1656.25
16	2406.25	2281.25	1843.75
17	2718.75	2562.50	2093.75
18	3062.50	2875.00	2343.75
19	3437.50	3250.00	2656.25
20	3812.50	3625.00	3000.00
21	4281.25	4031.25	3406.25
22	4750.00	4500.00	3875.00
23	5281.25	5031.25	4406.25
24	5875.00	5537.50	5093.75
25	6531.25	6187.50	5937.50
26	7218.75	6875.00	6906.25

**Table 2:** Evaluation of the RF model with MFCC, HFCC and BFCC filters, by individually employing the first four moments of their distributions (mean-μ, standard deviation-STD, skewness-skew and kurtosis-kurt), their Δ and their ΔΔ. 10-cross-validation with 20,000 laughs (18,000 training and 2000 test in 10 epochs). AR, accuracy rate; TP, true positive; FP, false positive; TN, true negative; FN, false negative; Sens, sensitivity; Spec, specificity. Best performance per column is highlighted in bold.

Results by employing μ, STD, skewness and kurtosis of the coefficients								
Inputs	AR (%)	TP	FP	TN	FN	Sens	Spec	AUC
μ(MFCC)	72	0.72	0.28	0.68	0.32	0.69	0.71	0.722
STD(MFCC)	68	0.67	0.33	0.69	0.31	0.68	0.67	0.695
skew(MFCC)	59	0.58	0.42	0.61	0.39	0.6	0.59	0.615
kurt(MFCC)	60	0.62	0.38	0.59	0.41	0.6	0.61	0.625
μ(HFCC)	72	0.72	0.28	0.69	0.32	0.7	0.71	0.725
STD(HFCC)	70	0.7	0.3	0.69	0.31	0.7	0.7	0.721
skew(HFCC)	65	0.65	0.35	0.65	0.35	0.65	0.65	0.67
kurt(HFCC)	70	0.71	0.29	0.68	0.32	0.69	0.7	0.715
μ(BFCC)	73	0.72	0.28	0.7	0.3	0.71	0.71	0.733
STD(BFCC)	70	0.7	0.3	0.69	0.31	0.69	0.69	0.712
skew(BFCC)	57	0.57	0.43	0.58	0.42	0.57	0.57	0.599
kurt(BFCC)	63	0.65	0.35	0.62	0.39	0.63	0.63	0.654
**Results by employing μ, STD, skewness and kurtosis of the delta (Δ) of the coefficients**								
Inputs	AR (%)	TP	FP	TN	FN	Sens	Spec	AUC
μ(Δ(MFCC))	67	0.69	0.31	0.65	0.35	0.66	0.68	0.692
STD(Δ(MFCC))	74	0.7	0.3	0.65	0.35	0.66	0.68	0.694
skew(Δ(MFCC))	64	0.65	0.35	0.64	0.36	0.64	0.65	0.665
kurt(Δ(MFCC))	62	0.64	0.36	0.6	0.4	0.62	0.63	0.645
μ(Δ(HFCC))	69	0.7	0.3	0.68	0.32	0.69	0.69	0.712
STD(Δ(HFCC))	70	0.68	0.32	0.67	0.33	0.67	0.68	0.695
skew(Δ(HFCC))	70	0.7	0.3	0.71	0.29	0.7	0.7	0.72
kurt(Δ(HFCC))	64	0.67	0.33	0.61	0.39	0.63	0.65	0.664
μ(Δ(BFCC))	63	0.65	0.35	0.62	0.38	0.63	0.64	0.657
STD(Δ(BFCC))	71	0.68	0.32	0.69	0.31	0.69	0.69	0.71
skew(Δ(BFCC))	68	0.69	0.31	0.67	0.33	0.68	0.68	0.701
kurt(Δ(BFCC))	63	0.66	0.35	0.6	0.4	0.62	0.64	0.656
**Results by employing μ, STD, skewness and kurtosis of the delta-delta (ΔΔ) of the coefficients**								
Inputs	AR (%)	TP	FP	TN	FN	Sens	Spec	AUC
μ(ΔΔ(MFCC))	69	0.72	0.28	0.65	0.35	0.68	0.7	0.712
STD(ΔΔ(MFCC))	71	0.78	0.22	0.75	0.25	0.71	0.72	0.735
skew(ΔΔ(MFCC))	61	0.8	0.2	0.77	0.23	0.61	0.61	0.634
kurt(ΔΔ(MFCC))	66	0.79	0.21	0.77	0.23	0.66	0.66	0.685
μ(ΔΔ(HFCC))	69	0.71	0.29	0.66	0.34	0.68	0.7	0.713
STD(ΔΔ(HFCC))	71	0.73	0.27	0.69	0.31	0.7	0.72	0.734
skew(ΔΔ(HFCC))	66	0.65	0.35	0.66	0.34	0.66	0.65	0.675
kurt(ΔΔ(HFCC))	61	0.63	0.37	0.6	0.4	0.61	0.62	0.635
μ(ΔΔ(BFCC))	63	0.65	0.35	0.62	0.38	0.63	0.64	0.655
STD(ΔΔ(BFCC))	73	0.74	0.26	0.73	0.27	0.73	0.73	0.754
skew(ΔΔ(BFCC))	70	0.7	0.3	0.69	0.31	0.69	0.7	0.715
kurt(ΔΔ(BFCC))	60	0.58	0.42	0.62	0.38	0.6	0.6	0.626

**Table 3:** Evaluation of the RF model with MFCC, HFCC and BFCC filters, by incrementally employing the first four moments of their distributions (mean-μ, standard deviation-STD, skewness-skew and kurtosis-kurt), their Δ and their ΔΔ. 10-cross-validation with 20,000 laughs (18,000 training and 2000 test in 10 epochs). AR, accuracy rate; TP, true positive; FP, false positive; TN, true negative; FN, false negative; Sens, sensitivity; Spec, specificity. Best performance per column is highlighted in bold.

Results by employing μ, STD, skewness and kurtosis of the coefficients								
Inputs	AR (%)	TP	FP	TN	FN	Sens	Spec	AUC
μ(MFCC)	72	0.72	0.28	0.68	0.32	0.69	0.71	0.71
μ+STD(MFCC)	74	0.75	0.25	0.73	0.27	0.73	0.74	0.75
μ+STD+skew(MFCC)	75	0.76	0.24	0.74	0.26	0.74	0.75	0.76
μ+STD+skew+kurt(MFCC)	76	0.77	0.23	0.76	0.24	0.76	0.77	0.78
μ(HFCC)	72	0.72	0.28	0.69	0.31	0.70	0.71	0.72
μ+STD(HFCC)	74	0.74	0.26	0.73	0.27	0.74	0.74	0.76
μ+STD+skew(HFCC)	76	0.77	0.23	0.75	0.25	0.76	0.76	0.78
μ+STD+skew+kurt(HFCC)	77	0.79	0.21	0.76	0.24	0.77	0.78	0.80
μ(BFCC)	73	0.72	0.28	0.70	0.30	0.71	0.71	0.73
μ+STD(BFCC)	74	0.75	0.25	0.73	0.27	0.73	0.74	0.76
μ+STD+skew(BFCC)	75	0.76	0.24	0.74	0.26	0.75	0.75	0.77
μ+STD+skew+kurt(BFCC)	76	0.77	0.23	0.75	0.25	0.76	0.76	0.79
**Results by employing μ, STD, skewness and kurtosis of the delta (Δ) of the coefficients**								
Inputs	AR (%)	TP	FP	TN	FN	Sens	Spec	AUC
μ(Δ(MFCC))	67	0.69	0.31	0.65	0.35	0.66	0.68	0.69
μ+STD(Δ(MFCC))	72	0.73	0.27	0.72	0.28	0.72	0.73	0.75
μ+STD+skew(Δ(MFCC))	73	0.75	0.25	0.72	0.28	0.73	0.74	0.76
μ+STD+skew+kurt(Δ(MFCC))	75	0.76	0.24	0.75	0.25	0.75	0.76	0.78
μ(Δ(HFCC))	69	0.7	0.3	0.68	0.32	0.69	0.69	0.71
μ+STD(Δ(HFCC))	72	0.71	0.29	0.72	0.28	0.72	0.71	0.74
μ+STD+skew(Δ(HFCC))	73	0.73	0.27	0.73	0.27	0.73	0.73	0.75
μ+STD+skew+kurt(Δ(HFCC))	76	0.76	0.24	0.76	0.24	0.76	0.76	0.78
μ(Δ(BFCC))	63	0.65	0.35	0.62	0.38	0.63	0.64	0.66
μ+STD(Δ(BFCC))	67	0.67	0.33	0.68	0.32	0.67	0.67	0.69
μ+STD+skew(Δ(BFCC))	69	0.69	0.31	0.70	0.30	0.69	0.69	0.71
μ+STD+skew+kurt(Δ(BFCC))	71	0.72	0.28	0.72	0.28	0.72	0.72	0.74
**Results by employing μ, STD, skewness and kurtosis of the delta-delta (ΔΔ) of the coefficients**								
Inputs	AR (%)	TP	FP	TN	FN	Sens	Spec	AUC
μ(ΔΔ(MFCC))	69	0.72	0.28	0.65	0.35	0.68	0.70	0.71
μ+STD(ΔΔ(MFCC))	76	0.78	0.22	0.75	0.25	0.76	0.77	0.79
μ+STD+skew(ΔΔ(MFCC))	78	0.79	0.21	0.77	0.23	0.78	0.79	0.81
μ+STD+skew+kurt(ΔΔ(MFCC))	78	0.80	0.20	0.77	0.23	0.78	0.79	0.81
μ(ΔΔ(HFCC))	69	0.71	0.29	0.66	0.34	0.68	0.70	0.71
μ+STD(ΔΔ(HFCC))	75	0.76	0.24	0.73	0.27	0.74	0.75	0.77
μ+STD+skew(ΔΔ(HFCC))	75	0.77	0.24	0.74	0.26	0.75	0.76	0.78
μ+STD+skew+kurt(ΔΔ(HFCC))	76	0.77	0.23	0.75	0.25	0.75	0.77	0.78
μ(ΔΔ(BFCC))	63	0.65	0.35	0.62	0.38	0.63	0.64	0.66
μ+STD(ΔΔ(BFCC))	72	0.73	0.27	0.72	0.28	0.72	0.72	0.74
μ+STD+skew(ΔΔ(BFCC))	73	0.73	0.27	0.73	0.27	0.73	0.73	0.75
μ+STD+skew+kurt(ΔΔ(BFCC))	74	0.75	0.26	0.74	0.26	0.74	0.74	0.76

**Table 4:** Evaluation of the RF model with MFCC, HFCC and BFCC filters, by incrementally employing the first four moments of their distributions (mean-μ, standard deviation-STD, skewness-skew and kurtosis-kurt), together with their Δ and their ΔΔ. 10-cross-validation with 20,000 laughs (18,000 training and 2000 test in 10 epochs). AR, accuracy rate; TP, true positive; FP, false positive; TN, true negative; FN, false negative; Sens, sensitivity; Spec, specificity. Best performance per column is highlighted in bold.

Inputs	AR (%)	TP	FP	TN	FN	Sens	Spec	AUC
μ(MFCC+Δ(MFCC)+ΔΔ(MFCC))	74	0.77	0.23	0.71	0.29	0.73	0.76	0.75
μ+STD(MFCC+Δ(MFCC)+ΔΔ(MFCC))	82	0.83	0.17	0.82	0.18	0.82	0.83	0.84
μ+STD+skew(MFCC+Δ(MFCC)+ΔΔ(MFCC))	83	0.84	0.16	0.82	0.18	0.82	0.84	0.85
μ+STD+skew+kurt(MFCC+Δ(MFCC)+ΔΔ(MFCC))	83	0.84	0.16	0.82	0.18	0.83	0.84	0.86
μ(HFCC+Δ(HFCC)+ΔΔ(HFCC))	75	0.77	0.23	0.73	0.27	0.74	0.76	0.76
μ+STD(HFCC+Δ(HFCC)+ΔΔ(HFCC))	81	0.82	0.18	0.81	0.19	0.81	0.82	0.83
μ+STD+skew(HFCC+Δ(HFCC)+ΔΔ(HFCC))	82	0.83	0.17	0.82	0.18	0.82	0.82	0.84
μ+STD+skew+kurt(HFCC+Δ(HFCC)+ΔΔ(HFCC))	82	0.83	0.17	0.82	0.18	0.82	0.83	0.85
μ(BFCC+Δ(BFCC)+ΔΔ(BFCC))	72	0.74	0.26	0.70	0.30	0.74	0.71	0.76
μ+STD(BFCC+Δ(BFCC)+ΔΔ(BFCC))	80	0.80	0.20	0.80	0.20	0.80	0.80	0.82
μ+STD+skew(BFCC+Δ(BFCC)+ΔΔ(BFCC))	81	0.81	0.19	0.81	0.19	0.81	0.81	0.84
μ+STD+skew+kurt(BFCC+Δ(BFCC)+ΔΔ(BFCC))	82	0.82	0.18	0.81	0.19	0.82	0.81	0.85

**Table 5:** Results of the variation of the kernel in the SVM model with MFCC, HFCC and BFCC filters, by employing the first four moments of their distributions (mean-μ, standard deviation-STD, skewness-skew and kurtosis-kurt). 10-cross-validation with 20,000 laughs (18,000 training and 2000 test in 10 epochs). AR, accuracy rate; TP, true positive; FP, false positive; TN, true negative; FN, false negative; Sens, sensitivity; Spec, specificity. Best performance per column is highlighted in bold.

Results of Mel filters: μ + STD + skew + kurt (MFCC + Δ(MFCC) + ΔΔ(MFCC))								
Kernel variation	AR (%)	TP	FP	TN	FN	Sens	Spec	AUC
Linear	74	0.74	0.26	0.73	0.27	0.73	0.74	0.76
Polynomial	73	0.75	0.25	0.72	0.28	0.73	0.74	0.76
Radial Basis	65	0.86	0.14	0.45	0.55	0.61	0.76	0.72
ν-Linear	81	0.81	0.19	0.81	0.19	0.81	0.81	0.85
ν-Polynomial	82	0.82	0.18	0.83	0.17	0.82	0.82	0.86
ν-Radial Basis	73	0.85	0.15	0.60	0.40	0.68	0.80	0.79
**Results of Human Factor filters: μ + STD + skew + kurt (HFCC + Δ(HFCC) + ΔΔ(HFCC))**								
Kernel variation	AR (%)	TP	FP	TN	FN	Sens	Spec	AUC
Linear	74	0.74	0.26	0.73	0.27	0.74	0.74	0.78
Polynomial	74	0.75	0.25	0.73	0.27	0.73	0.74	0.78
Radial Basis	66	0.86	0.14	0.45	0.55	0.61	0.76	0.73
ν-Linear	81	0.81	0.19	0.81	0.19	0.81	0.81	0.85
ν-Polynomial	83	0.83	0.17	0.83	0.17	0.83	0.83	0.86
ν-Radial Basis	73	0.85	0.15	0.61	0.39	0.69	0.81	0.79
**Results of Bark filters: μ + STD + skew + kurt (BFCC + Δ(BFCC) + ΔΔ(BFCC))**								
Kernel variation	AR (%)	TP	FP	TN	FN	Sens	Spec	AUC
Linear	71	0.71	0.29	0.72	0.28	0.72	0.71	0.76
Polynomial	72	0.72	0.28	0.72	0.28	0.72	0.72	0.76
Radial Basis	63	0.85	0.15	0.41	0.59	0.59	0.73	0.69
ν-Linear	80	0.80	0.20	0.80	0.20	0.80	0.80	0.85
ν-Polynomial	82	0.82	0.18	0.82	0.18	0.82	0.82	0.86
ν-Radial Basis	66	0.85	0.15	0.47	0.53	0.62	0.76	0.72

**Table 6:** Summary of the results of the RF model with MFCC, HFCC and BFCC filters, by employing the first four moments of their distributions (mean-μ, standard deviation-STD, skewness-skew and kurtosis-kurt), Δ and ΔΔ. 10-cross-validation with 20,000 laughs (18,000 training and 2000 test in 10 epochs). AR, accuracy rate; TP, true positive; FP, false positive; TN, true negative; FN, false negative; Sens, sensitivity; Spec, specificity. Note that the three rows correspond to the 4th, 8th and 12th row of Table 4.

	AR (%)	TP	FP	TN	FN	Sens	Spec	AUC
MFCC	83	0.84	0.16	0.82	0.18	0.83	0.84	0.86
HFCC	82	0.83	0.17	0.82	0.18	0.82	0.83	0.85
BFCC	81	0.82	0.18	0.81	0.19	0.82	0.81	0.85

## Data Availability

Not applicable.
